# Parkin ubiquitination of Kindlin-2 enables mitochondria-associated metastasis suppression

**DOI:** 10.1016/j.jbc.2023.104774

**Published:** 2023-05-02

**Authors:** Minjeong Yeon, Irene Bertolini, Ekta Agarwal, Jagadish C. Ghosh, Hsin-Yao Tang, David W. Speicher, Frederick Keeney, Khalid Sossey-Alaoui, Elzbieta Pluskota, Katarzyna Bialkowska, Edward F. Plow, Lucia R. Languino, Emmanuel Skordalakes, M. Cecilia Caino, Dario C. Altieri

**Affiliations:** 1 Immunology, Microenvironment and Metastasis Program, The Wistar Institute, Philadelphia, Pennsylvania, USA; 2 Proteomics and Metabolomics Shared Resource, The Wistar Institute, Philadelphia, Pennsylvania, USA; 3 Molecular and Cellular Oncogenesis Program, The Wistar Institute, Philadelphia, Pennsylvania, USA; 4 Imaging Shared Resource, The Wistar Institute, Philadelphia, Pennsylvania, USA; 5 Department of Medicine, Case Comprehensive Cancer Center, Case Western Reserve University, Cleveland, Ohio, USA; 6 Department of Cardiovascular and Metabolic Sciences, Lerner Research Institute, Cleveland Clinic, Cleveland, Ohio, USA; 7 Department of Cancer Biology, Sidney Kimmel Cancer Center, Thomas Jefferson University, Philadelphia, Pennsylvania, USA; 8 Gene Expression and Regulation Program, The Wistar Institute, Philadelphia, Pennsylvania, USA; 9 Department of Pharmacology, University of Colorado School of Medicine, Aurora, Colorado, USA

**Keywords:** Parkin, tumor cell motility, Kindlin-2, ubiquitination, metastasis suppression

## Abstract

Mitochondria are signaling organelles implicated in cancer, but the mechanisms are elusive. Here, we show that Parkin, an E3 ubiquitination (Ub) ligase altered in Parkinson’s disease, forms a complex with the regulator of cell motility, Kindlin-2 (K2), at mitochondria of tumor cells. In turn, Parkin ubiquitinates Lys581 and Lys582 using Lys48 linkages, resulting in proteasomal degradation of K2 and shortened half-life from ∼5 h to ∼1.5 h. Loss of K2 inhibits focal adhesion turnover and β1 integrin activation, impairs membrane lamellipodia size and frequency, and inhibits mitochondrial dynamics, altogether suppressing tumor cell–extracellular matrix interactions, migration, and invasion. Conversely, Parkin does not affect tumor cell proliferation, cell cycle transitions, or apoptosis. Expression of a Parkin Ub-resistant K2 Lys581Ala/Lys582Ala double mutant is sufficient to restore membrane lamellipodia dynamics, correct mitochondrial fusion/fission, and preserve single-cell migration and invasion. In a 3D model of mammary gland developmental morphogenesis, impaired K2 Ub drives multiple oncogenic traits of EMT, increased cell proliferation, reduced apoptosis, and disrupted basal–apical polarity. Therefore, deregulated K2 is a potent oncogene, and its Ub by Parkin enables mitochondria-associated metastasis suppression.

Mitochondria are invariably exploited in cancer (1, 2). This involves adaptive changes in bioenergetics important for tumor growth (3, 4) but also a plethora of other functions in redox balance (5); control of cell death (6); modulation of mitochondrial size, shape, and subcellular distribution, *i.e.*, mitochondrial dynamics (7, 8); and interorganelle cross talk (9) that enables key disease traits, including heightened cell motility and invasion (10).

Aside from the roles in tumor promotion, there is now emerging evidence that mitochondria may also participate in the opposite function of tumor suppression, specifically targeting cancer cell movements (11). Accordingly, ablation of disparate regulators of mitochondrial dynamics (12), bioenergetics (13) or mitophagy (14) enhances tumor cell invasion and metastasis (7). Molecules that selectively inhibit tumor cell movements without affecting other cancer traits have been described (15), but whether such metastasis suppressors involve mitochondria is unknown, and mechanistic link(s) between mitochondria and the signaling machinery of cell motility (16) have not been clearly delineated.

A potential regulator of mitochondria-associated tumor suppression is Parkin, an E3 ubiquitination (Ub) ligase and effector of mitophagy (17) altered in early-onset Parkinson’s disease (18) and lost in virtually all human cancers (19). How Parkin antagonizes tumorigenesis, and whether this involves mitophagy (20), has remained unclear and variously linked to metabolic rewiring (21), regulation of mitosis (22), changes in iron homeostasis (23) and/or inhibition of serine metabolism (24). However, a recent Ub screen carried out in the absence of harsh and potentially nonphysiologic mitochondria-depolarizing conditions (25) suggested a different scenario and identified Parkin as a potent inhibitor of mitochondria-fueled tumor cell movements (26). This pathway required Parkin E3 ligase activity but was independent of mitophagy (26).

In this context, Kindlins have emerged as important effectors of cell motility, including in cancer (27). These molecules are members of the 4.1-ezrin-ridixin-moesin (FERM) domain containing proteins and the three mammalian members of this family, Kindlin-1 (K1 or FERMT1), Kindlin-2 (K2 or FERMT2), and Kindlin-3 (K3 or FERMT3), regulate membrane dynamics of cell movements (28), integrin-dependent cell–extracellular matrix (ECM) interactions (29) and cell spreading and migration (30).

Here, we identified K2 as a target of Parkin Ub, enabling a novel pathway of mitochondria-associated metastasis suppression.

## Results and discussion

### K2 is a novel substrate of Parkin Ub

**Figure 1:**
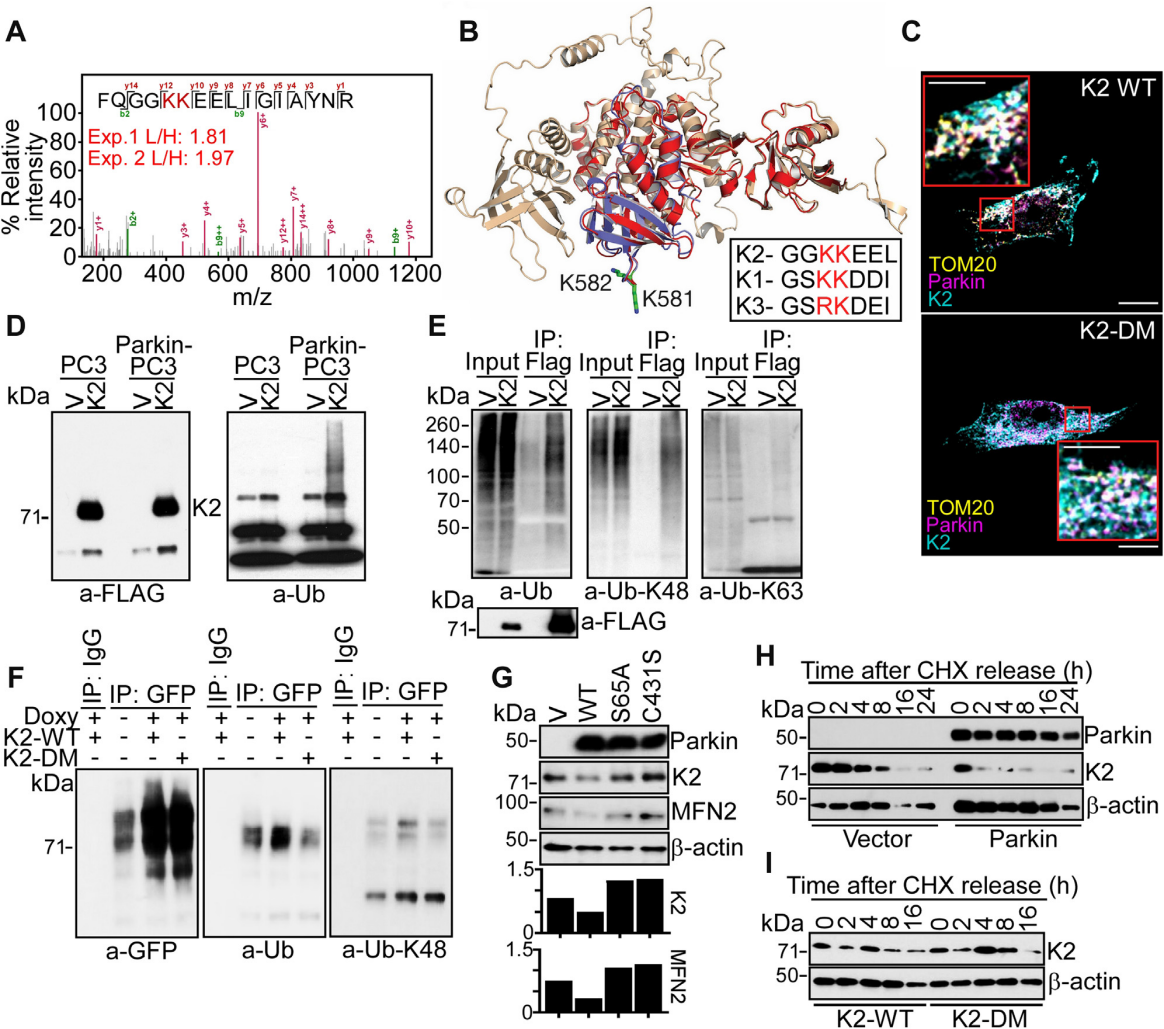
**Parkin-K2 regulation at mitochondria.***A*, MS/MS spectrum of triply charged *m/z* 646.3459 ion corresponding to the K2 ubiquitinated peptide FQGGKKEELIGIAYNR. The SILAC labeling ratio (L, Parkin; H, Vector) is indicated. The predicted Parkin Ub sites in K2, K581, and K582 are shown. *B*, AlphaFold model of human K2 (RMSD between K2 and K3, 1.2 Å). The position of K581 and K582 is indicated. *Inset*, sequence alignment of K family proteins. *C*, PC3 cells expressing Parkin were reconstituted with K2-WT or K2-DM and imaged by confocal microscopy. TOM20 was a mitochondrial marker. Merged images are shown. The scale bar represents 10 μm. *Inset*, magnification of indicated areas. The scale bar represents 5 μm. *D*, parental or Parkin-expressing PC3 cells (Parkin-PC3) were transfected with FLAG-vector (V) or FLAG-K2, and immune complexes precipitated with an antibody to FLAG were analyzed for expression of FLAG (*left*) or ubiquitin (Ub, *right*) by Western blotting. *E*, FLAG-K2 immune complexes as in (*D*) were analyzed with an antibody to total Ub or K63 or K48 Ub by Western blotting. *F*, PC3 cells with Doxy-induced conditional expression of Parkin were reconstituted with GFP-K2-WT or GFP-K2-DM, and immune complexes precipitated with IgG or an antibody to GFP were analyzed for reactivity with GFP (*left*), Ub (*middle*), or K48 Ub (*right*) by Western blotting. *G*, PC3 cells expressing vector (V), WT Parkin, or Parkin S65A or C431S mutant were analyzed by Western blotting. *Bottom*, densitometric quantification of protein bands. *H*, PC3 cells as in (*D*) were analyzed by cycloheximide (CHX) block and release followed by Western blotting at the indicated time intervals. *I*, Parkin-expressing PC3 cells were reconstituted with K2-WT or K2-DM and analyzed by CHX block and release and Western blotting. Doxy, doxycycline; MS/MS, tandem mass spectroscopy; SILAC, stable isotope labeling by amino acids in cell culture.

To identify physiologic targets of Parkin in cancer, a Ub screen was carried out in the absence of mitochondria-depolarizing conditions (25) using stable isotope labeling by amino acids in cell culture (SILAC) (26). A top hit in the screen was K2, which exhibited a two-fold increase in SILAC labeling in the presence of Parkin, compared with vector (Fig. 1*A*). The tandem mass spectroscopy spectrum of the triply charged *m/z* 646.3459 ion identified the K2 peptide FQGGKKEELIGIAYNR, with both K581 and K582 as candidate Ub sites for Parkin (Fig. 1*A*). In a structural model of the -COOH terminus FERM (F3) domain of K2, both K581 and K582 are surface-exposed (Fig. 1*B*), upstream of Q(614)W and other functionally relevant sites in K2 (reviewed in the study by Plow and Quin (27)). By sequence alignment, K582 was conserved in all 3 K-family proteins, whereas K581 was conserved in K1 and K2 and substituted to R in K3 (Fig. 1*B*, inset). On the other hand, only K2 was identified as a potential Parkin Ub substrate in our SILAC screen (26). Next, we asked if Parkin interacted with K2 in cancer. Immune complexes of Myc–Parkin expressed in PC3 cells contained GFP-K2 (Fig. S1*A*). Reciprocally, K2 immunoprecipitates were associated with Parkin (Fig. S1*B*). IgG immune complexes were unreactive (Fig. S1, *A* and *B*). Consistent with these data, Parkin and K2 exhibited extensive colocalization at mitochondria, by confocal fluorescence microscopy (Figs. 1*C* and S1*C*). To test whether the two candidate Ub sites were important for this interaction, we next generated a K2 K581A/K582A double mutant (DM) that abolishes Parkin-predicted Ub sites (Fig. S1*D*). In these experiments, both K2-WT and K2-DM were comparably associated with Parkin at mitochondria, by confocal microscopy (Fig. 1*C*; Fig. S1, *C* and *E*).

## Parkin Ub regulates K2 stability

**Figure 2:**
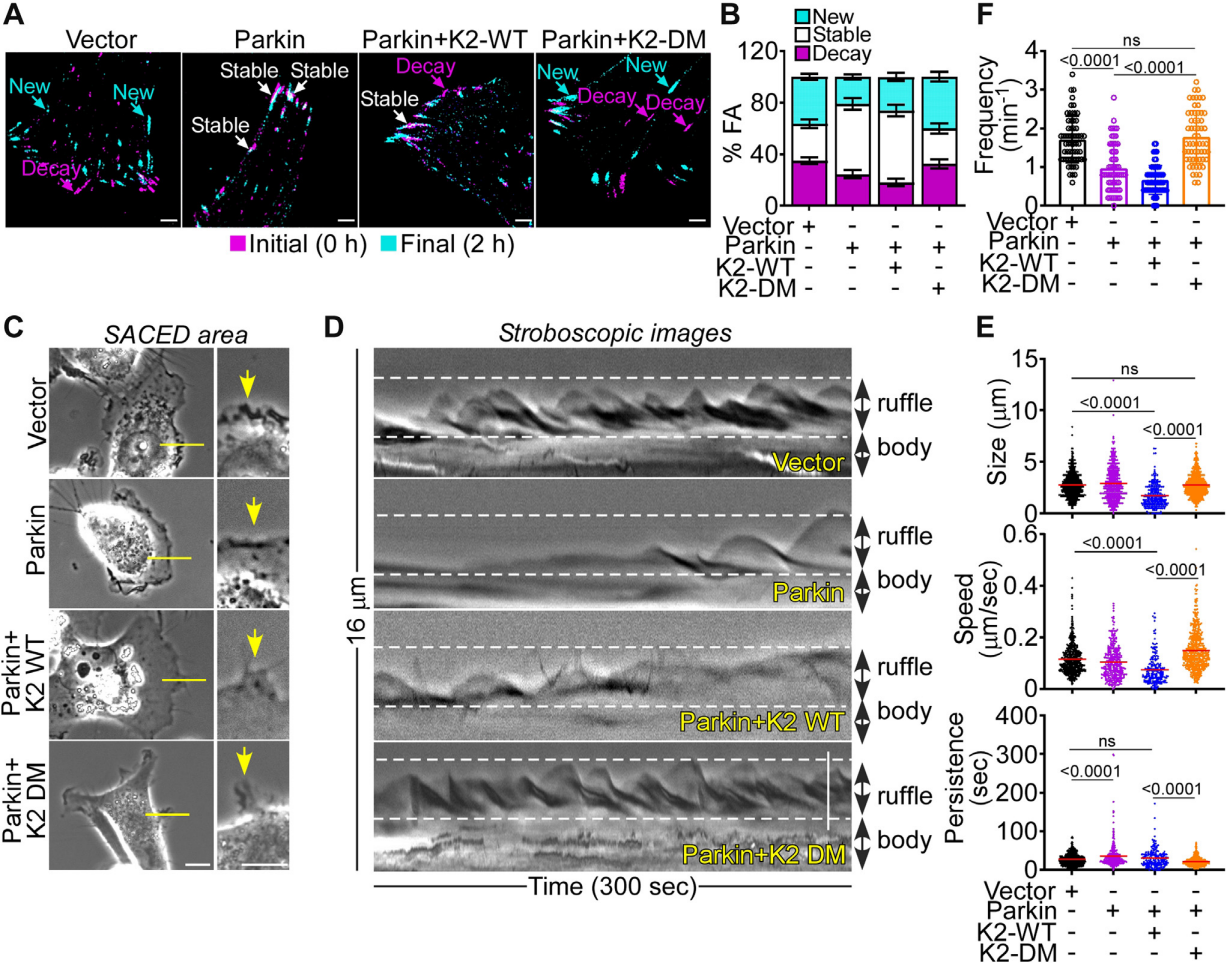
**Modulation of plasma membrane dynamics.***A* and *B*, Parkin-expressing LN229 cells were reconstituted with K2-WT or K2-DM, labeled with Talin-GFP and imaged by time-lapse videomicroscopy (*A*) with quantification of stable, decay, and new FA (*B*). Representative merged frames at 0 h (*magenta*) and 2 h (*cyan*) are shown. The scale bar represents 15 μm. Mean ± SEM (n = 17–19). The statistics are as follows: new FA, vector vs. Parkin, *p* = 0.009; vector *versus* Parkin + K2-WT, ns; vector *versus* Parkin + K2-DM, ns; stable FA, vector *versus* Parkin, *p* < 0.0001; vector *versus* Parkin + K2-WT, *p* < 0.0001; vector *versus* Parkin + K2-DM, ns; decay FA, vector *versus* Parkin, ns; vector *versus* Parkin + K2-WT, *p* = 0.003; vector *versus* Parkin + K2-DM, ns. *C*, LN229 cells as in (*A*) were analyzed by real-time phase-contrast imaging. Representative images show individual cells and one area examined for membrane dynamics (*arrows*). The scale bar represents 10 μm. *D*, cell membrane dynamics for each discrete area in (*C*) is represented as a stroboscopic image, where the cell body and ruffle/lamellae are labeled. The scale bar represents 10 μm. *E*, each individual cell protrusion was analyzed for lamella size (*top*), speed of retraction (*middle*), and lamella persistence (*bottom*). Each point represents an individual membrane protrusion with 203 (Parkin + K2-WT), 299 (Parkin), 513 (vector), and 532 (Parkin + K2-DM) membrane events from 15 individual cells per group. Mean values per group are indicated. *F*, the conditions are as in (*E*), and the frequency of lamella dynamics was quantified. Mean ± SD (n = 60). For all panels, numbers correspond to *p* values by one-way ANOVA with Tukey multiple comparisons test. ns, not significant.

K2, but not vector, precipitated from Parkin-expressing PC3 cells reacted with an antibody to total Ub (Fig. 1*D*), as well as K48 Ub, whereas K63 Ub was unreactive (Fig. 1*E*). To further characterize this pathway, we next generated clones of PC3 cells with conditional expression of Parkin (TET-ON system) induced by treatment with doxycycline (Doxy) (Fig. S1*F*). Immune complexes of K2-DM precipitated from Doxy-induced Parkin-expressing cells showed reduced reactivity for total Ub and K48 Ub, compared with K2-WT (Fig. 1*F*). Consistent with Ub-mediated protein degradation, transient (Fig. S2*A*) or stable (Fig. S2*B*) expression of Parkin in tumor cells resulted in loss of endogenous K2 levels. Conversely, Parkin Ub-resistant K2 mutants, K581A, K582A, or K2-DM, were not affected (Fig. S2*C*). As an independent approach, we expressed Parkin E3 ligase–defective mutant S65A or C431S in PC3 cells (Fig. 1*G*). These mutants did not reduce the levels of endogenous K2 or mitofusin-2, a known target of Parkin Ub, whereas both molecules were lost in the presence of wildtype (WT) Parkin (Fig. 1*G*). Finally, we carried out cycloheximide block-and-release experiments to quantify how Parkin Ub regulates K2 stability. Expression of WT Parkin shortened the half-life (t½) of K2 from ∼5 h to ∼1.5 h in PC3 cells (Figs. 2*H* and S2*D*). In contrast, K2-DM half-life was not affected (Figs. 1*I* and Fig. S2*E*).

### Regulation of plasma membrane dynamics by Parkin-K2 Ub

K2 regulates plasma membrane dynamics of cell–ECM interactions (29) and cell motility (28) and the role of Parkin Ub in this process was next investigated. Consistent with previous observations (26), Parkin expression inhibited focal adhesion (FA) turnover, which is essential for cell motility (16), reducing the fraction of new and decayed FA while expanding the population of stable FA (Fig. 2, *A* and *B*). This was associated with impaired membrane lamellipodia dynamics (Fig. 2, *C* and *D*), another requirement of cell movements (31). In these experiments, Parkin expression decreased the lamella size and speed of lamella retraction while prolonging lamella persistence at the plasma membrane (Figs. 2*E* and S3*A*). The frequency of lamella formation was also inhibited in these settings, by single-cell Stroboscopic Analysis of Cell Dynamics (SACED) quantification (Figs. 2*F* and S3*B*). A third upstream requirement of tumor cell movements is heightened mitochondrial dynamics (32). Reconstitution of tumor cells with Parkin inhibited this process (26), reducing the rate of both organelle fusion and fission events (Fig. S4, *A* and *B*). Based on these collective results, we asked if K2 Ub contributed to Parkin suppression of plasma membrane and mitochondrial dynamics in the control of cell movements. Accordingly, reconstitution of Parkin-expressing PC3 cells with K2-DM, but not K2-WT, restored the kinetics of FA turnover (Fig. 2, *A* and *B*), maintained membrane lamellipodia dynamics (Fig. 2, *C*–*F*; Fig. S3, *A* and *B*), and rescued mitochondrial fusion and fission events (Fig. S4, *A* and *B*), when compared to controls. Reconstitution with Ub-resistant single K2 K581A or K582A mutant also rescued mitochondrial dynamics in the presence of Parkin (Fig. S4*B*).

### Parkin-K2 regulation of tumor cell–ECM interaction and cell motility

**Figure 3:**
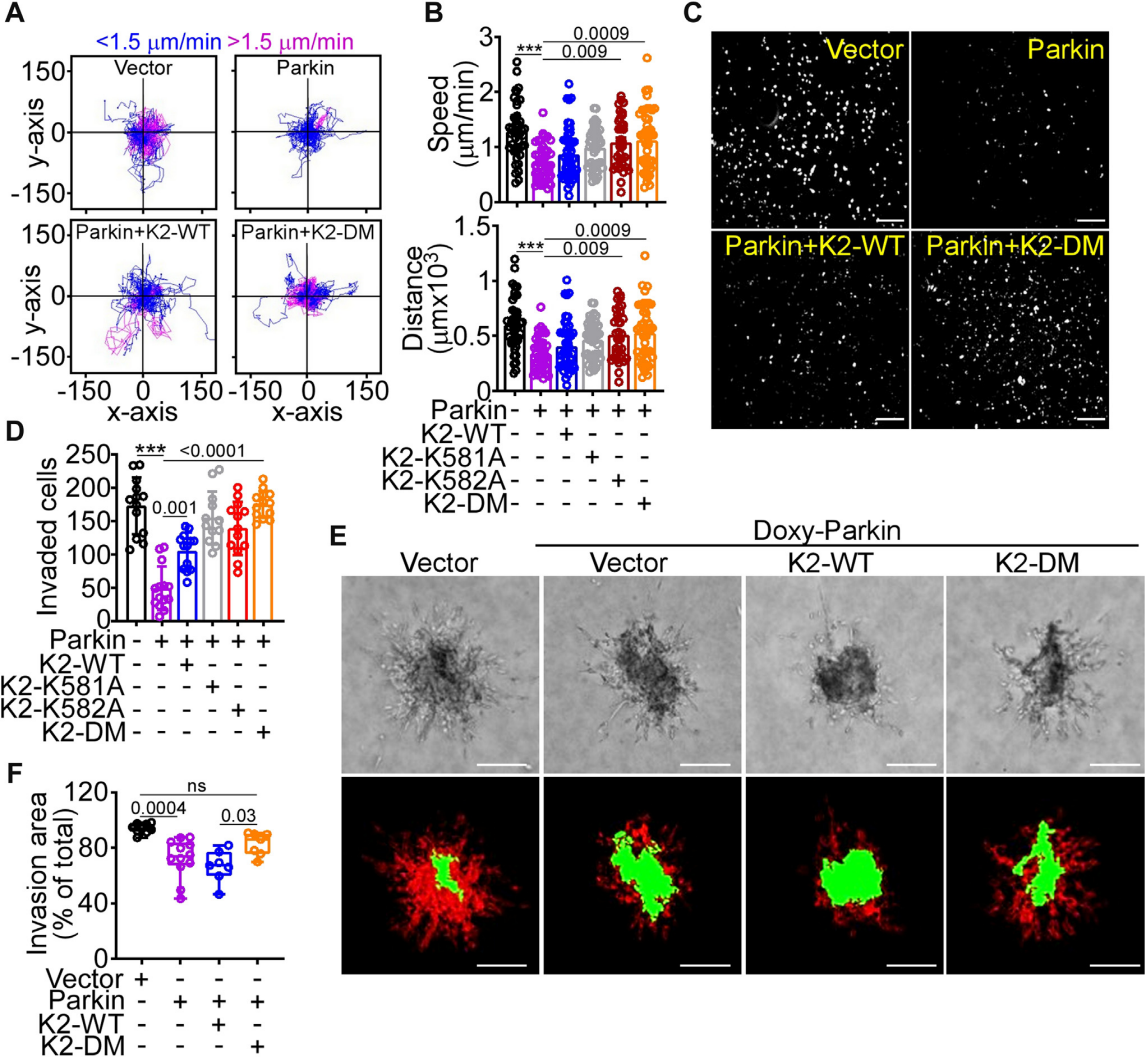
**Regulation of tumor cell migration and invasion.***A* and *B*, PC3 cells expressing vector or Parkin were reconstituted with K2-WT or K2-DM and analyzed for single-cell motility by time-lapse videomicroscopy in 2D contour plots (*A*), and the average speed of cell movements (*B*, *top*) and total distance traveled by individual cells (*B*, *bottom*) was quantified. Each tracing corresponds to an individual cell. The cutoff velocities for slow (*blue*)- or fast (*purple*)-moving cells are indicated. Mean ± SD (n = 34–46). ∗∗∗*p* < 0.0001. *C* and *D*, PC3 cells as in (*A*) were analyzed for invasion across Matrigel-coated Transwell inserts and DAPI-stained nuclei of invaded cells (*C*, representative images) were quantified (*D*). The scale bar represents 200 μm. Mean ± SD (n = 12–14). ∗∗∗*p* < 0.0001. *E*, PC3 cells with Doxy-induced Parkin expression were reconstituted with K2-WT or K2-DM, seeded in 3D spheroids in a collagen-containing matrix, and cell invasion was visualized by light microscopy (*top*) and INSIDIA software image reconstruction (*bottom*) for quantification of core area (*green*) and invasive area (*red*). The scale bar represents 200 μm. *F*, the conditions are as in (*E*), and the invasion area of 3D spheroids was quantified (min to max; n = 7–9). For all panels, numbers correspond to *p* values by one-way ANOVA with Tukey's multiple comparisons test. DAPI, 4′,6-diamidino-2-phenylindole; Doxy, doxycycline.

**Figure 4:**
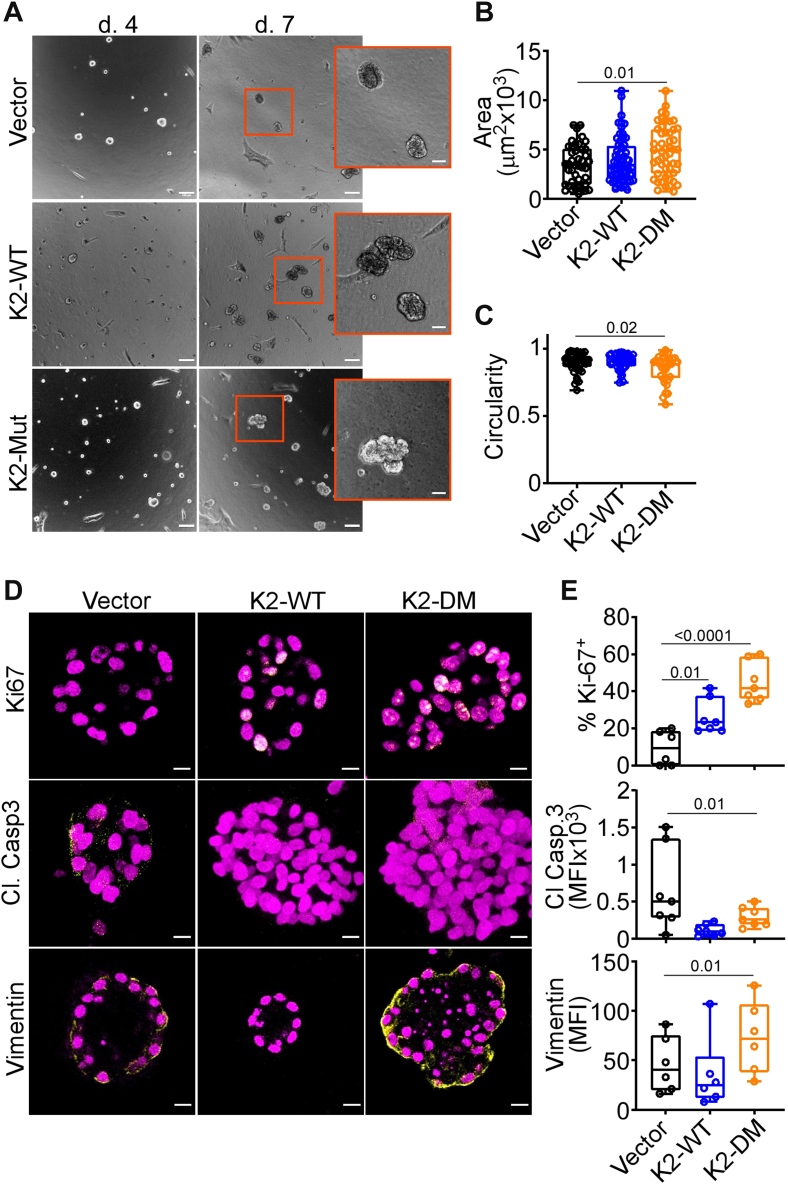
**Oncogenic properties of deregulated K2.***A*, normal breast epithelial MCF10A cells were transfected with K2-WT or K2-DM, embedded in 2% Matrigel, and 3D acini were imaged by bright-field microscopy after 4 and 7 days. Representative images. The scale bar represents 100 μm. *Insets*, magnification of indicated areas. The scale bar represents 50 μm. *B* and *C*, the conditions are as in (*A*), and 3D acini surface area (*B*) and circularity (*C*) were quantified (mean to max, n = 33–60). *D* and *E*, Three-dimensional acini as in (*A*) were harvested after 8 days or 14 days (vimentin) and analyzed by immunofluorescence microscopy (*D*, representative images) with quantification of the percentage of Ki-67 (*E*, *top*)-, cleaved (Cl) caspase-3 (*E*, *middle*)-, and vimentin (*E*, *bottom*)-positive cells. MFI, mean fluorescence intensity (min to Max, n = 6–7). The scale bar represents 10 μm. For all panels, numbers correspond to *p* values by one-way ANOVA with Tukey multiple comparisons test.

Consistent with inhibition of membrane dynamics, Parkin expression inhibited tumor cell adhesion (Fig. S5*A*) and spreading (Fig. S5, *B* and *C*) onto the ECM substrate, fibronectin. This was accompanied by impaired activation of β1 integrin (Fig. S5*D*), a process regulated by K2 (29), whereas total β1 integrin levels were unchanged (Fig. S5*E*). In addition, Parkin expression inhibited single-cell motility (26), reducing the speed of tumor cell movements and the total distance traveled by individual cells (Fig. 3, *A* and *B*). Tumor cell invasion across Matrigel-coated inserts (Fig. 3, *C* and *D*), as well as 3D invasion of tumor spheroids embedded in collagen (Fig. 3, *E* and *F*), was also inhibited in these settings. Conversely, reconstitution of Parkin-expressing cells with K2-DM or K2 mutants K581A or K582A restored tumor cell adhesion (Fig. S5*A*) and spreading (Fig. S5, *B* and *C*), rescued single-cell movements (Fig. 3, *A* and *B*), and enabled tumor cell invasion across Matrigel inserts (Fig. 3, *C* and *D*), as well as in 3D tumor spheroids (Fig. 3, *E* and *F*). Reconstitution with K2-WT had limited to no effect (Fig. S5, A–C; Fig. 4, *E* and *F*).

As an independent approach, expression of E3 ligase-defective Parkin S65A or C431S mutant (Fig. S6*A*) failed to reduce tumor cell adhesion to fibronectin or fibrinogen (Fig. S6*B*) and did not affect single-cell motility (26), compared with WT Parkin. Similarly, treatment with the proteasome inhibitor, MG132 prevented Parkin degradation of K2 in cycloheximide block-and-release experiments (Fig. S6*C*) and preserved the speed of tumor cell movements and total distance traveled by individual cells, when compared to controls (Fig. S6, *D* and *E*).

Finally, we asked if Parkin inhibition of tumor–ECM interactions and cell motility was specific. In these experiments, Parkin expression in PC3 cells had no effect on cell proliferation (Fig. S7*A*), DNA content (Fig. S7*B*), or distribution of cell cycle phases (Fig. S7*C*). Similarly, annexin V labeling, which quantifies apoptosis, was unchanged in control or Parkin-expressing cells (Fig. S7*D*).

### Oncogenic properties of deregulated K2

To test how a Parkin-K2 Ub axis may affect mechanisms of tumorigenesis, we next expressed K2-DM in normal, nontransformed breast epithelial MCF10A cells, which contain endogenous Parkin (26). In these experiments, non-Parkin ubiquitinatable K2 increased focal adhesion kinase phosphorylation (Tyr397); upregulated EMT markers, vimentin, and SNAIL (Fig. S8*A*); and heightened cell migration, compared with control (Fig. S8*B*). Expression of K2-WT had a more limited effect (Fig. S8, *A* and *B*). In a 3D model of mammary gland developmental morphogenesis, expression of K2-DM, but not K2-WT, increased the surface area of 3D acini (Fig. 4, *A* and *B*) and reduced acini circularity (Fig. 4*C*), two markers of deregulated epithelial morphogenesis. This was accompanied by increased cell proliferation, reduced apoptosis, and upregulation of EMT vimentin (Fig. 4, *D* and *E*). In addition, K2-DM disrupted apical–basal polarity in 3D acini, resulting in deregulated expression of apical (GM130) and basal (hDlg) polarity markers and aberrant filling of the acini lumen, by phalloidin staining (Fig. S8*C*).

In summary, we have identified Parkin Ub-dependent degradation of K2 (29) as a novel mechanism of mitochondria-associated metastasis suppression, shutting off multiple upstream requirements of cell–ECM interaction and cell motility (16). This is consistent with key roles of K2 in FA turnover (28), integrin activation (33) and signaling requirements of cell movements (28), exploited for tumor progression (34). Mechanistically, there is precedent for K2 regulation by Ub (35) and participation in mitochondrial functions (36), in line with the formation of a Parkin–K2 complex at mitochondria described here. On the other hand, a role of K581 and K582 or K48 Ub linkages in K2 stability has not been proposed (35). The Parkin-K2 axis uncovered here selectively inhibited tumor cell motility and invasion, without affecting other tumor traits of cell proliferation, cell cycle, or apoptosis. This selectivity is a hallmark of metastasis suppressors (15), identifying Parkin as a novel, mitochondria-associated antagonist of metastasis. In this context, the nearly universal loss of Parkin in cancer (19) is expected to impair K2 Ub and proteasomal degradation. This enables potent oncogenic properties of unbridled K2 in normal epithelia, disrupting tissue morphogenesis and heightening tumor cell motility and invasion to promote metastasis.

## Experimental procedures

### Cells and cell cultures

Prostate adenocarcinoma PC3 and DU145, neuroblastoma SK-N-SH (SKN), glioblastoma LN229, NIH3T3 fibroblasts, and normal breast epithelial MCF10A cells were obtained from the American Type Culture Collection. Cell line authentication was carried out as described (26). PC3 clones with conditional expression of Parkin (TET-ON system) were generated using plasmid pLV[Exp]-CMV>tTS/rtTA/Hygro and pLV[Exp]-Pruo-TRE>hPRKN with lentivirus particles (multiplicity of infection 10) generated from VectorBuilder. Clones established in puromycin (1 μg/ml) were transduced with tTS/rtTA-encoding lentiviruses, selected in hygromycin B (100 μg/ml), and conditional Parkin expression was induced by Doxy (0.1 μg/ml) treatment.

### Antibodies and reagents

Antibodies to Parkin, GM130, Ki-67, cleaved caspase 3, vimentin, focal adhesion kinase, Tyr397-phosphorylated focal adhesion kinase, total ubiquitin (Ub), and K63 or K48 Ub linkages were acquired from Cell Signaling. An antibody to mitochondrial TOM20 was from Proteintech. An antibody to hDlg was from Santa Cruz. Antibodies to FLAG, K2, and β-actin were from Sigma-Aldrich. Antibodies to activated (FITC-conjugated HUTS-4) or total β1 integrin were from EMD Millipore and BioLegend, respectively. MitoTracker Green, Phalloidin Alexa488, CellLight Mitochondria-RFP, BacMam 2.0, and secondary antibodies for immunofluorescence were from Molecular Probes.

### Plasmids and protein analysis

A cDNA for WT Parkin was from GeneCopoeia. E3 ligase loss-of-function Parkin Ser65Ala (S65A) and Cys431Ser (C431S) mutants were generated using PfuUltra II fusion HS DNA Polymerase (Agilent Technologies). Ub-defective K2 mutant Lys581Ala (K581A), Lys 582Ala (K582A), or DM Lys581Ala/Lys582Ala (K581A/K582A) were generated using Stratagene QuikChange II XL Site-Directed Mutagenesis kit (Agilent Technologies) and confirmed by DNA sequencing. Protein expression was assessed by Western blotting and visualized by chemiluminescence (26). *In vitro* Ub studies were carried out as described (37).

### Plasma membrane dynamics

FA turnover was quantified by time-lapse videomicroscopy (12). Plasma membrane lamellipodia dynamics was imaged using a 40× objective on a Nikon TE300 inverted time-lapse microscope, and lamella size, persistence, and frequency were quantified by SACED (38).

### Fluorescence microscopy

Mitochondrial dynamics was quantified using CellLight Mitochondria-RFP, BacMam 2.0-transduced cells using a Leica TCS SP8 X inverted laser scanning confocal microscope with a 63X 1.40NA oil objective (26). For each cell, the volume of mitochondria over time was analyzed in four different areas (∼10 mitochondria per area) in 3D images. Variations in mitochondrial volume were evaluated by fold-change over time: a fold-change >1.3 denoted a fusion event, and a fold-change <0.7 denoted a fission event. The averaged fission and fusion events in four different areas were used for each cell analyzed. Parkin-K2 colocalization at mitochondria was examined with an antibody to TOM20 by confocal fluorescence microscopy.

### Single-cell motility

Experiments were carried out using 2D chemotaxis chambers (Ibidi) and a gradient setup with NIH3T3-conditioned medium (26). Time-lapse videomicroscopy was performed over 10 h with 10-min intervals. At least 30 cells were tracked using the Manual Tracking plugin for ImageJ, and the tracking data from independent time-lapse experiments were pooled and exported into the Chemotaxis and Migration Tool v2.0 (Ibidi) to calculate the speed of cell movements and the total distance traveled by individual cells.

### Cell adhesion, spreading, and invasion

Cells (4 × 10^4^, 50% confluency) were starved for 1 h in a reduced serum medium, plated onto a fibronectin-coated (10 μg/ml) 96-well plate, fixed in 4% paraformaldehyde, and stained with crystal violet. Cell adhesion was quantified by absorbance at 570 nm. For analysis of cell spreading, cells seeded onto an Ibidi 8-well μ-Slide were imaged with a Nikon TE inverted microscope after 24 h, and the cell surface was quantified by ImageJ. Tumor cell invasion across Matrigel-coated 8-μm PET Transwell chambers (Corning) was quantified in the presence of an NIH3T3-conditioned medium as a chemoattractant (26). The 3D organotypic tumor spheroids embedded in a collagen matrix were quantified for maximum invasion distance and invasion area (38).

### MCF10A 3D acini formation

The 3D cultures of MCF10A cells (5 × 10^3^/well) were performed using μ-Slide 8-well chambers (Ibidi) coated with 40 μl of Growth Factor Reduced Matrigel (BD Biosciences) plus 2% Matrigel and 5 ng/ml epidermal growh factor (39). Bright-field images were collected using a TE300 inverted microscope (Nikon) with a 10× objective. MCF10A acini were fixed in 4% formalin and processed for immunohistochemistry or immunofluorescence (40). Nuclei were counterstained with Hoechst (1:500), and images were captured using a Leica TCS SP8 confocal laser scanning microscope.

### Statistical analysis

Data are expressed as mean ± SD or mean ± SEM of results from a minimum of three independent experiments. Unpaired, two-tailed Student *t* test was used for two-group analyses. For multiple-group comparisons, analysis of variance was used. Statistical analyses were performed using a GraphPad software package (Prism 9.0) for Windows. A *p* value of <0.05 was considered statistically significant.

## Data availability

All data are contained within the article. Requests for reagents should be addressed to DC Altieri (daltieri@wistar.org).

## Supporting information

This article contains supporting information.

## Conflict of interest

The authors declare that they have no conflicts of interest with the contents of this article.
