# Supplementary material for: Parkin ubiquitination of Kindlin-2 enables mitochondria-associated metastasis suppression
Source: J Biol Chem. 2023 May 2;299(6):104774. doi: 10.1016/j.jbc.2023.104774 (PMC10236456; doi:10.1016/j.jbc.2023.104774)
Supplement: Supporting Figures S1–S8 [file mmc1.docx]

**PARKIN UBIQUITINATION OF KINDLIN-2 ENABLES MITOCHONDRIA-ASSOCIATED METASTASIS SUPPRESSION**

Minjeong Yeon, Irene Bertolini, Ekta Agarwal, Jagadish Ghosh, Hsin-Yao Tang, David W. Speicher, Frederick Keeney, Khalid Sossey-Alaoui, Elzbieta Pluskota, Katarzyna Bialkowska, Edward F. Plow, Lucia R. Languino, Emmanuel Skordalakes, M. Cecilia Caino and Dario C. Altieri

**SUPPORTING INFORMATION**

Figures S1-S8

**SUPPLEMENTAL FIGURES**


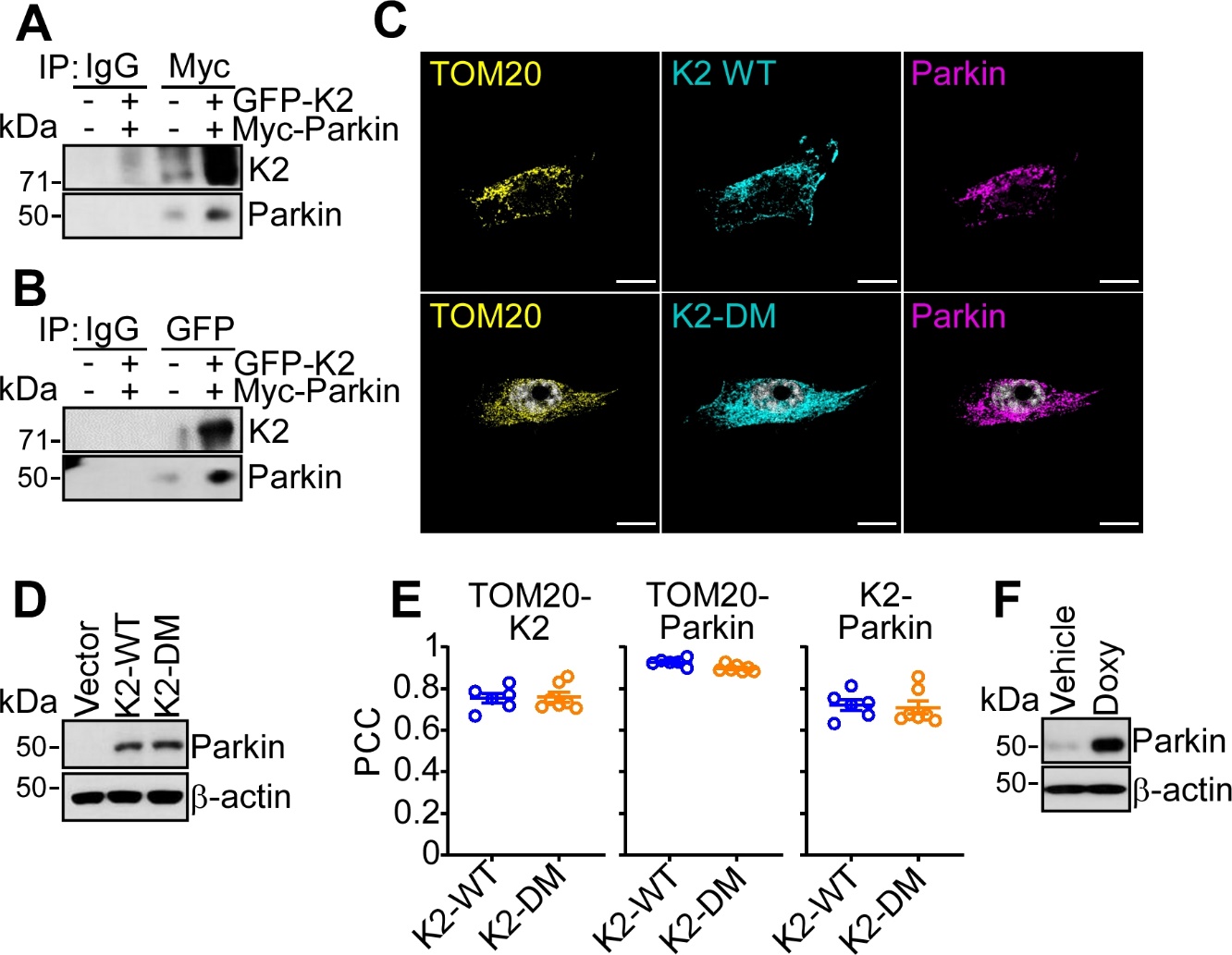


**Figure S1**. Parkin-K2 complex at mitochondria. A and B, PC3 cells transfected with Myc-Parkin were reconstituted with GFP-K2, immunoprecipitated (IP) with IgG or antibodies to Myc (A) or GFP (B) and immune complexes were analyzed by Western blotting. C, PC3 cells expressing Parkin were reconstituted with K2-WT or K2-DM and imaged for co-localization at mitochondria in the presence of an antibody to Tom20, by confocal fluorescence microscopy. Representative images are shown. Scale bars, 10 μm. Related to Fig. 1C. D, PC3 cells expressing K2-WT or K2-DM were analyzed by Western blotting. E, The conditions are as in (C) and mitochondrial co-localization of Parkin with K2-WT or K2-DM was quantified. TOM20 was a mitochondrial marker. Each point represents an individual determination. Mean±SEM (n=6-7). PCC, Pearson Correlation Coefficient. F, PC3 cells with conditional expression of Parkin in response to doxycycline (Doxy) were analyzed by Western blotting.


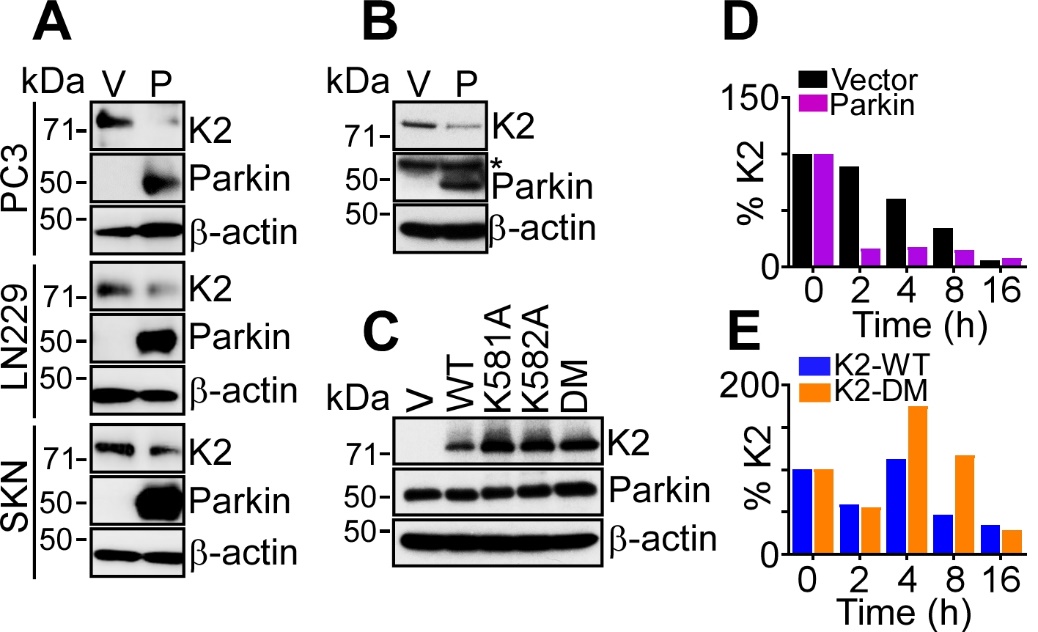


**Figure S2**. **Parkin regulation of K2 stability**. A, PC3, LN229 or SKN tumor cells were transfected with Parkin and analyzed by Western blotting. V, vector; P, Parkin. B, PC3 cells were stably transfected with vector (V) or Parkin (P) and analyzed by Western blotting. *, non-specific band. C, PC3 cells with stable expression of Parkin were transfected with vector (V), K2-WT, ubiquitination-resistant single K2 mutants, K581A, K582A or K2-DM and analyzed by Western blotting. D, PC3 cells expressing vector or Parkin were analyzed by cycloheximide (CHX) block and release followed by Western blotting and protein band quantification by densitometry at the indicated time intervals. E, Parkin-expressing PC3 cells reconstituted with K2-WT or K2-DM were analyzed by Western blotting at the indicated time intervals after CHX block and release and protein bands were quantified by densitometry.


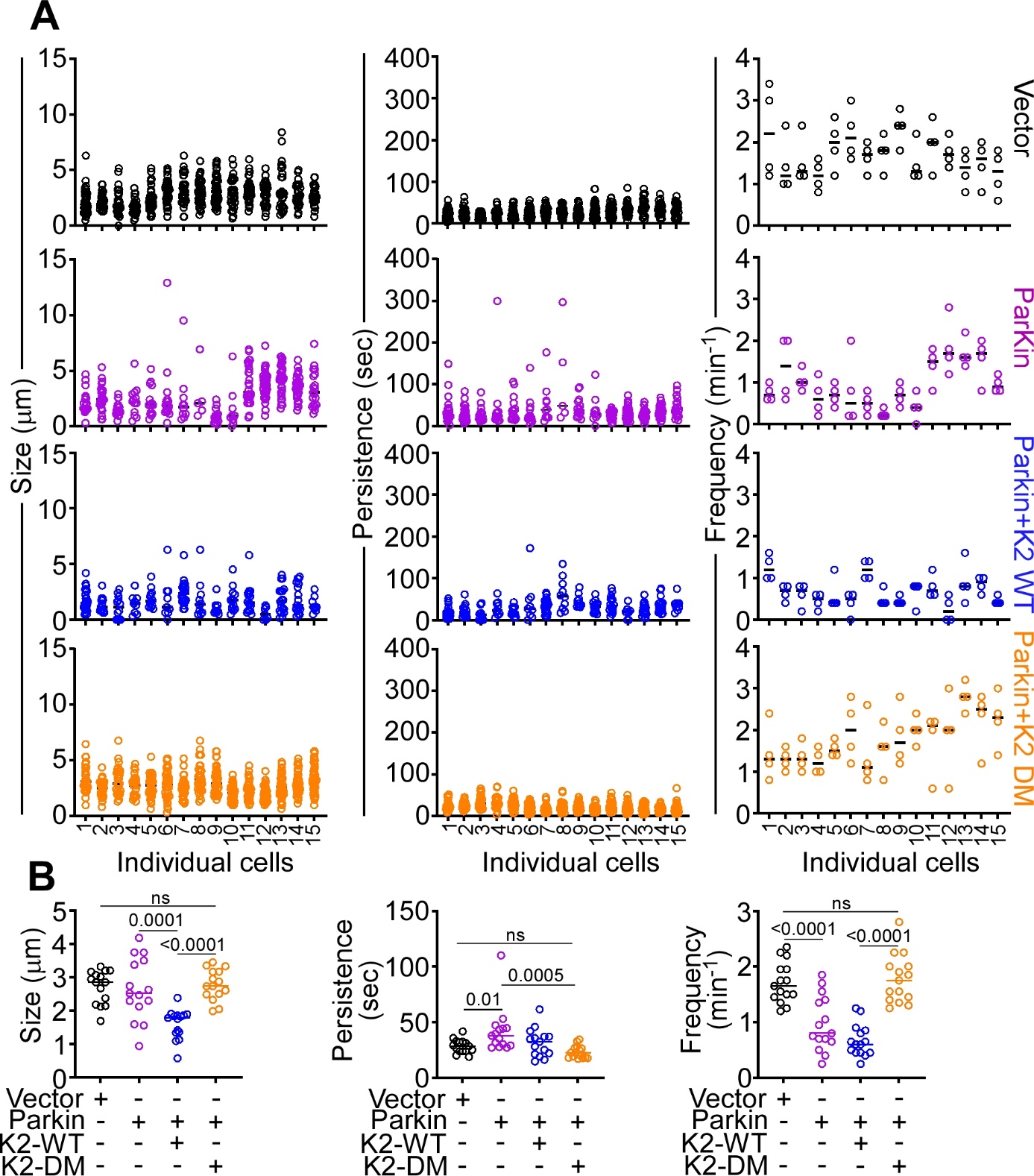


**Figure S3**. Parkin-K2 regulation of plasma membrane lamellipodia dynamics. A, LN229 cells expressing vector or Parkin were reconstituted with K2-WT or K2-DM and analyzed for kinetics of lamellipodia dynamics by time-lapse microscopy with quantification of changes in lamella size (*left*), persistence (*middle*) or frequency (*right*) in individual cells. Each point corresponds to analysis of an individual membrane protrusion (lamella size and persistence, n=24-47; lamella frequency, n=4). B, The conditions are as in (A) and the effect of K2-WT or K2-DM on Parkin inhibition of membrane lamellipodia dynamics per cell is represented as median (n=15). Numbers correspond to p values by one-way Anova with Tukey's multiple comparisons test.


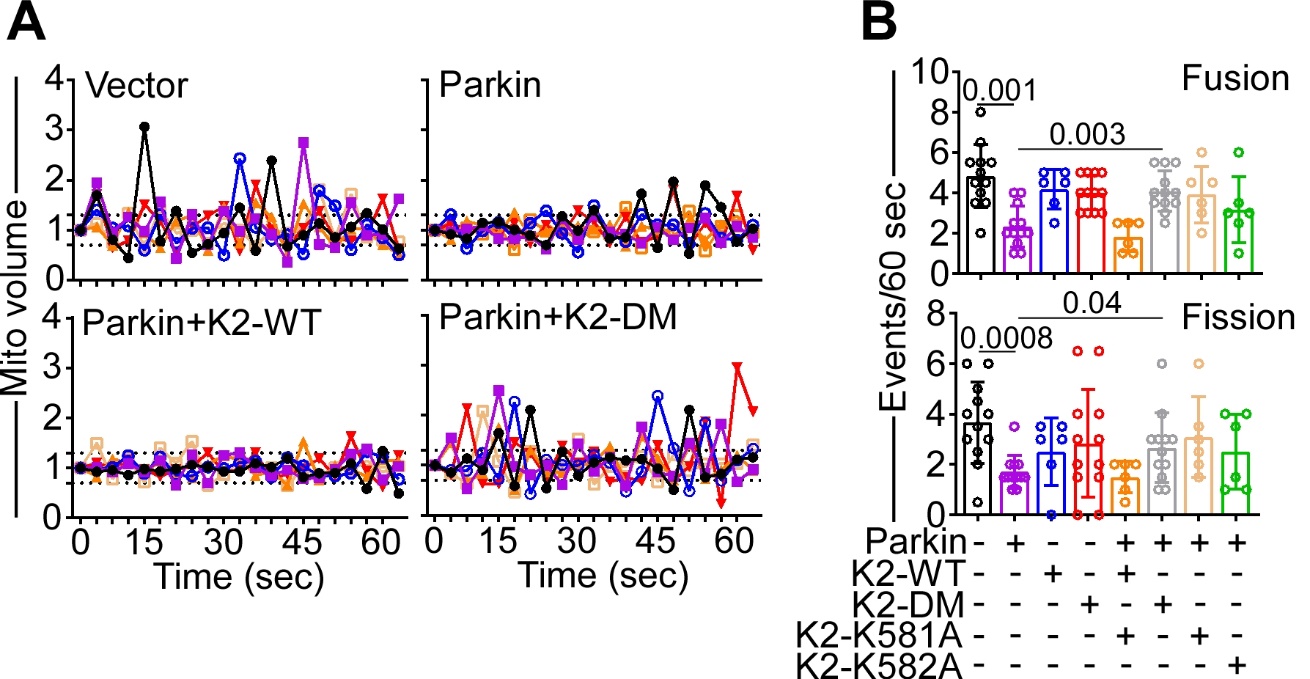


**Fig. S4. Parkin-K2 regulation of mitochondrial dynamics**. A, PC3 cells expressing vector or Parkin were reconstituted with K2-WT or K2-DM and analyzed for changes in mitochondrial volume by time-lapse videomicroscopy during a 60 sec interval. Each tracing corresponds to an individual cell. B, The conditions are as in (A) and mitochondrial fusion (>1.3-fold mitochondrial volume) and fission (<0.7-fold mitochondrial volume) events were quantified. Mean±SD (n=6-12). Numbers correspond to p values by one-way Anova with Tukey's multiple comparisons test.


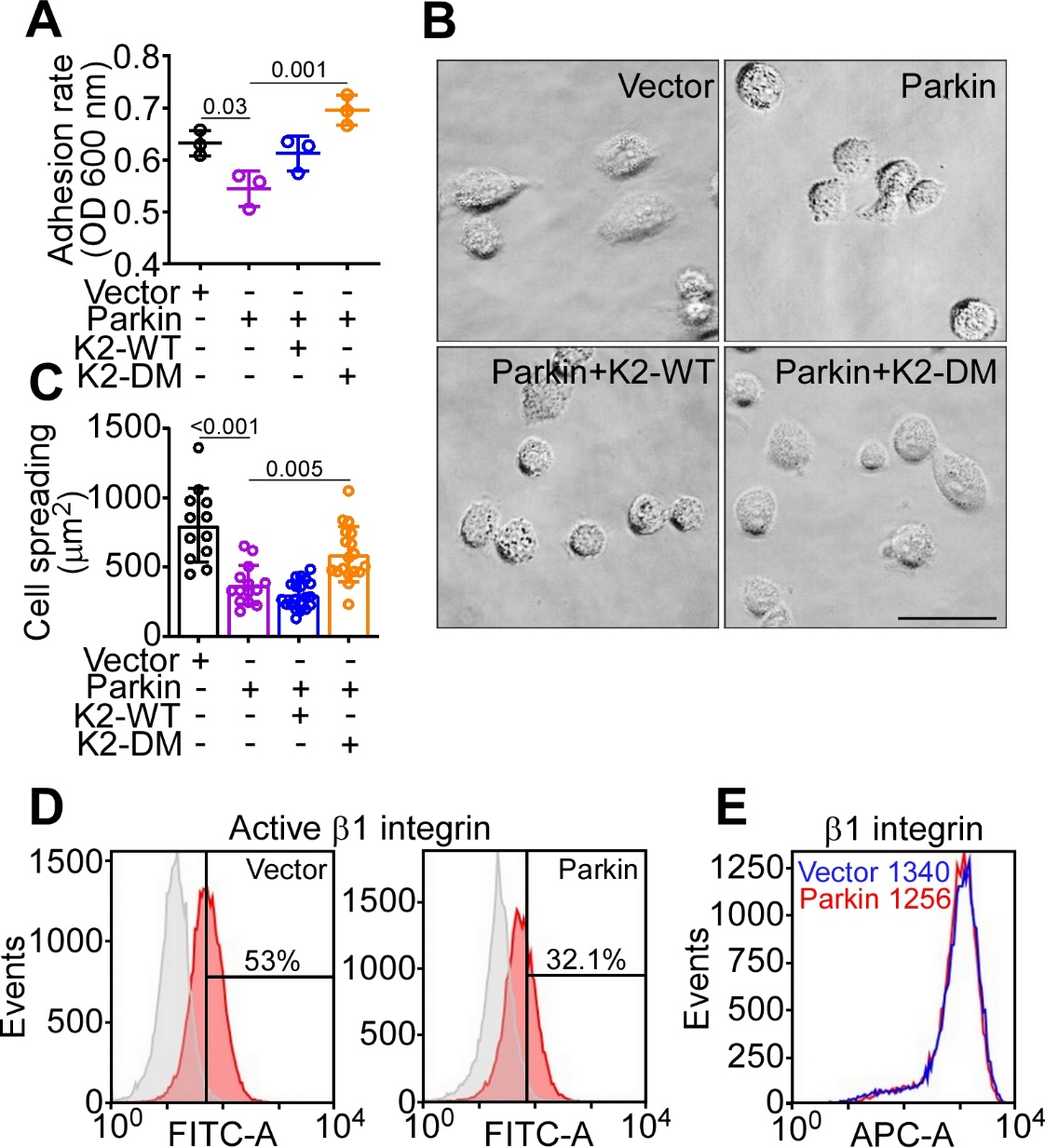


**Figure S5. Parkin-K2 control of tumor cell adhesion**. A, PC3 cells expressing Parkin were reconstituted with K2-WT or K2-DM and quantified for rate of adhesion to fibronectin. Mean±SD (n=3). B and C, The conditions are as in (A) and reconstituted PC3 cells were analyzed for spreading onto fibronectin-coated plates by light microscopy (B, representative images) and quantified (C). Scale bars, 50 μm. Mean±SD (n=12-20). Numbers correspond to p values by one-way Anova with Tukey's multiple comparisons test. D, PC3 cells expressing vector or Parkin were reconstituted with K2-DM and analyzed for changes in β1 integrin activation using an activation-dependent antibody by flow cytometry. The percentage of cells with β1 integrin activation is indicated (red profile). Grey profile, IgG staining. E, The conditions are as in (D) except that total β1 integrin expression was quantified by flow cytometry. Numbers indicate mean fluorescence units in cells expressing vector or Parkin.


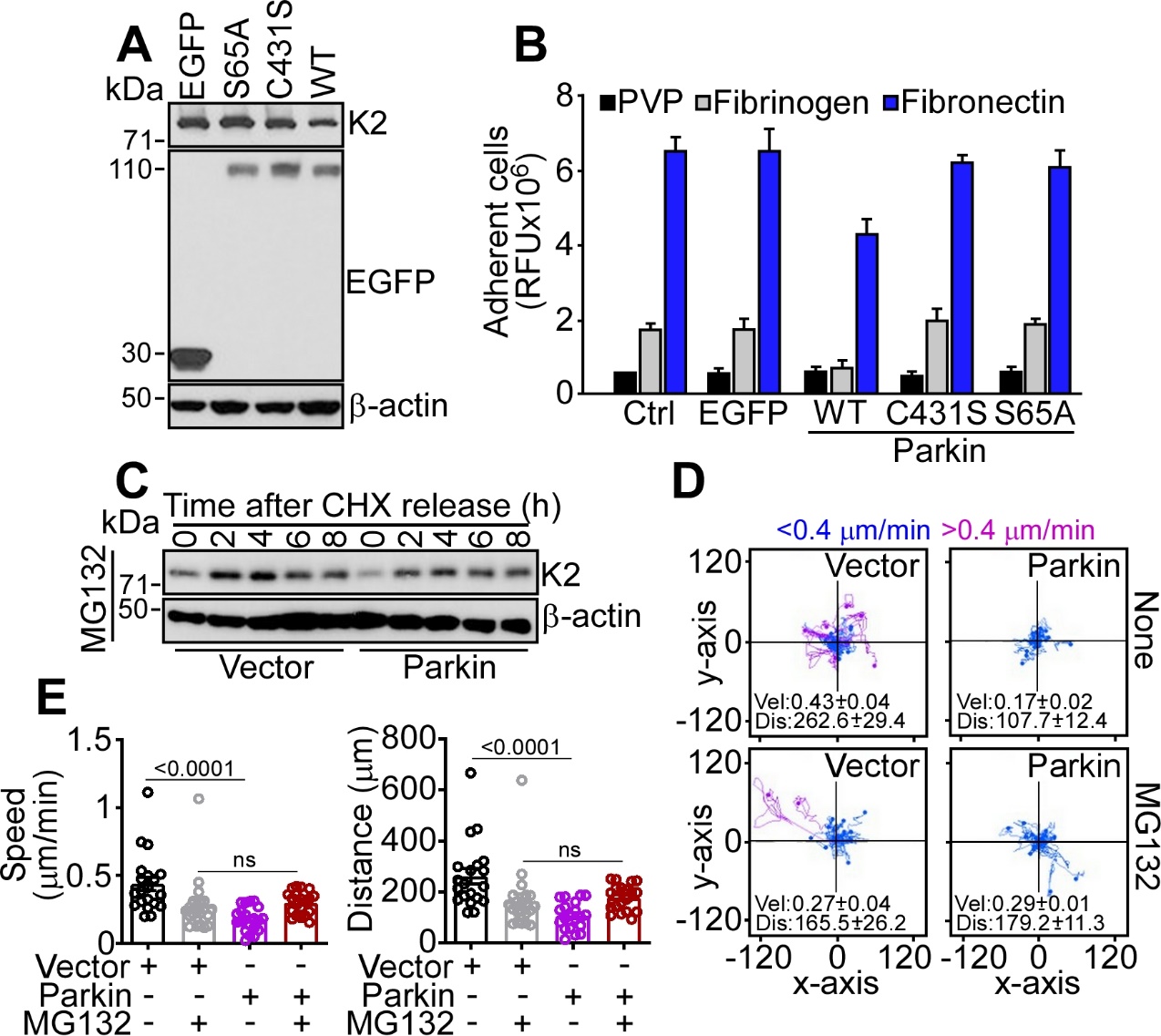


**Figure S6**. **Parkin E3 ligase-dependent regulation of cell adhesion and migration**. A, PC3 cells were transfected with WT Parkin or E3 Ub ligase defective Parkin S65A or C431S mutant fused to EGFP and analyzed by Western blotting. B, The conditions are as in (A) and transfected PC3 cells were analyzed for adhesion to fibrinogen- or fibronectin-coated plates. PVP was used as a control. Mean±SD. C, PC3 cells expressing vector or Parkin were analyzed for K2 stability in the presence of the proteasome inhibitor, MG132 by Western blotting at the indicated time intervals after cycloheximide (CHX) block and release. Representative experiment. D, PC3 cells expressing vector or WT Parkin were analyzed for single cell motility in the presence or absence of MG132 by time lapse videomicroscopy in 2D contour plots. Each tracing corresponds to an individual cell. The cutoff velocities for slow (blue)- or fast (purple)-moving cells are indicated. The average speed (Velocity, Vel, μm/min) and distance traveled (Dis, μm) was quantified per each condition. Mean±SD (n=20). E, The conditions are as in (D) and the speed of cell motility (*left*) and distance traveled (*right*) was quantified in each individual cell examined. Numbers correspond to p values by one-way Anova with Tukey's multiple comparisons test.


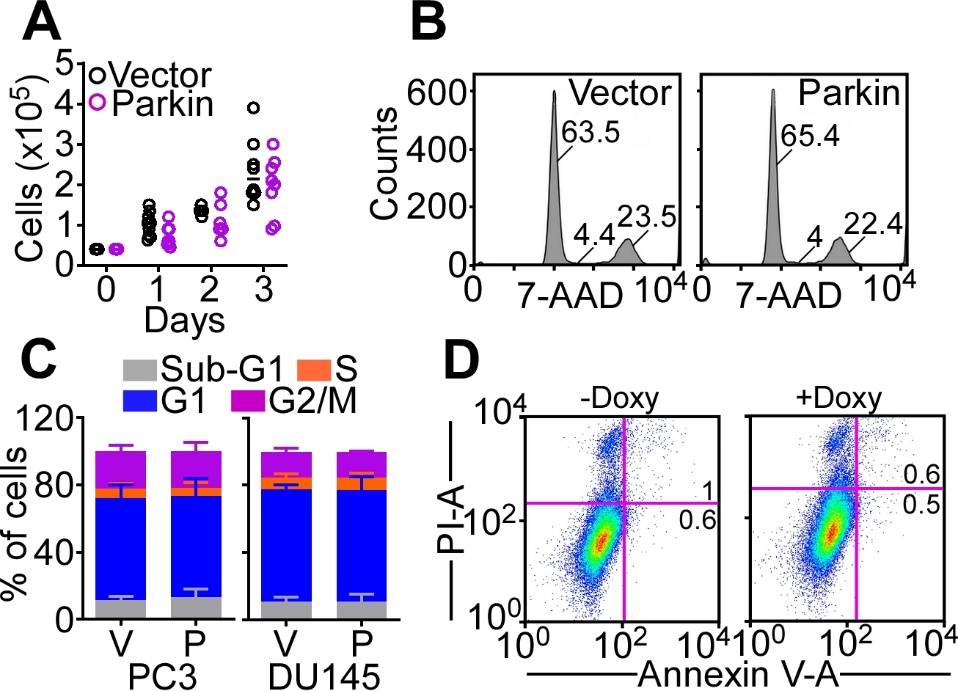


**Figure S7**. **Selectivity of Parkin tumor suppression function**. A, PC3 cells expressing Parkin or vector were analyzed for cell proliferation at the indicated time intervals by direct cell counting. B, PC3 cells as in (A) were analyzed for DNA content profile by propidium iodide (PI) staining and flow cytometry. The percentage of cells in the individual cell cycle phases is indicated. Representative experiment. C, PC3 or DU145 cells expressing Parkin (P) or vector (V) were analyzed for the indicated cell cycle phases by PI staining and flow cytometry. Average of two independent experiments. D, PC3 cells with or without doxycycline (Doxy)-induced conditional expression of Parkin were analyzed for Annexin V labeling and multiparametric flow cytometry. The percentage of cells in the indicated quadrants is indicated. Representative experiment.


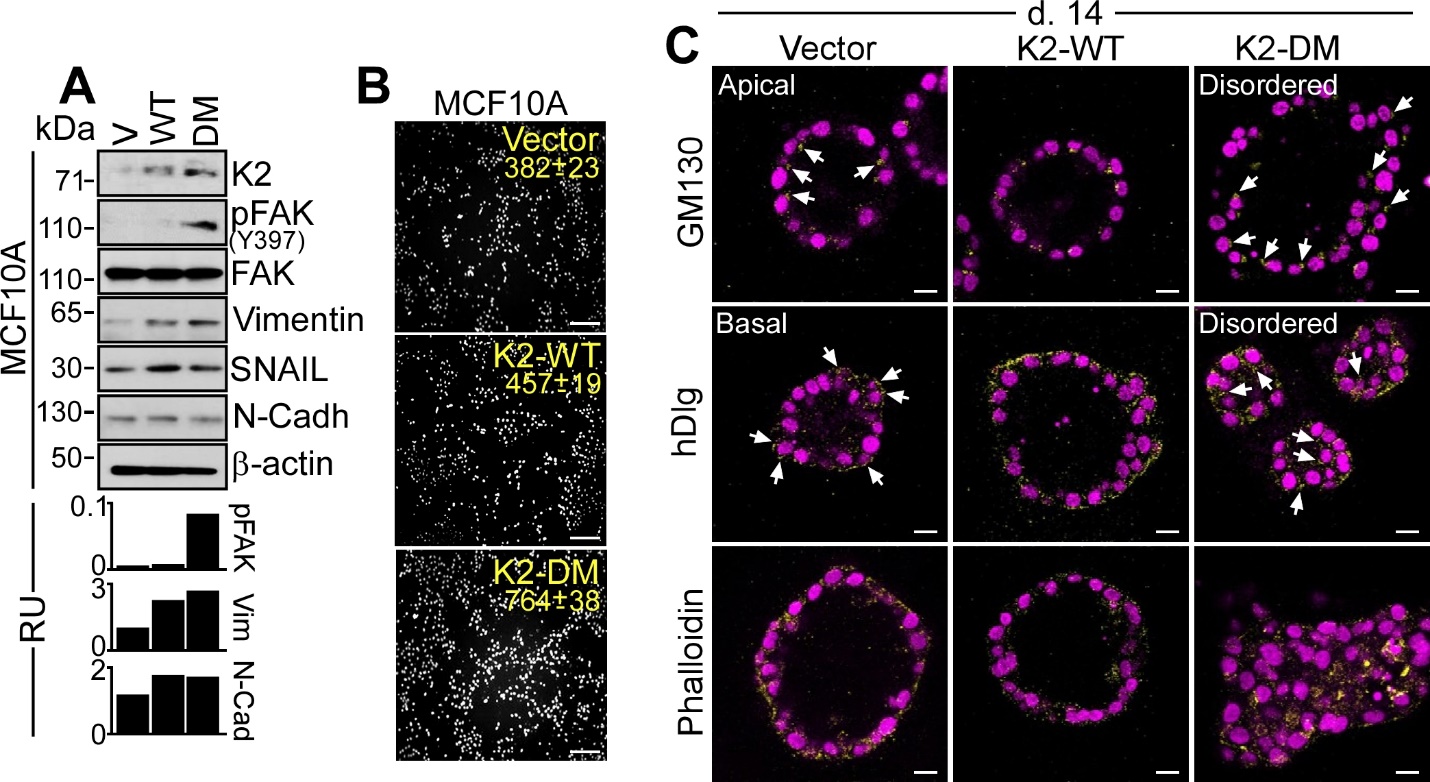


**Fig. S8**. **Deregulated K2-mediated oncogenesis**. A, Normal MCF10A breast epithelial cells expressing vector (V), K2-WT or K2-DM were analyzed by Western blotting with densitometric quantification of protein bands (*bottom*). N-Cadh, N-cadherin; Vim, vimentin. p, phosphorylation. RU, relative units. B, The conditions are as in (A) and transfected MCF10A cells were examined for migration on Transwell inserts and DAPI-stained nuclei of migrated cells were quantified. Scale bars, 200 μm. Mean±SEM (n=6), p<0.0001. C, MCF10A cells expressing vector, K2-WT or K2-DM were seeded in 3D acini in 2% Matrigel, harvested after 14 d and examined for differential distribution of apical (GM130) or basal (hDlg) polarity markers as well as phalloidin staining, by immunofluorescence microscopy. Scale bars, 10 μm. *Arrows*, disordered localization of apical and basal polarity markers. Representative images.
